# The influences of surface effect and elastic strain energy on structure and mechanical properties of dislocations in several diamond- and sphalerite-structured materials

**DOI:** 10.1371/journal.pone.0288331

**Published:** 2023-07-07

**Authors:** Huili Zhang

**Affiliations:** Department of Mathematics and Physics, Hebei Petroleum University of Technology, Chengde, China; Mohanlal Sukhadia University, INDIA

## Abstract

The fundamental properties of dislocations in diamond-structured Si and sphalerite-structured GaAs, InP and CdTe are investigated based on lattice theory of dislocation, hoping to provide some theoretical references in improving the properties of related materials. The influences of the surface effect(SE) and elastic strain energy on the structure and mechanical property of dislocation are discussed systematically. After considering the SE, the core width of dislocation becomes wider due to the elastic interaction between atoms becomes stronger. Compared to glide partial dislocation, the correction of SE to shuffle dislocation is more obvious. Both the SE and the elastic strain energy affect the energy barrier and Peierls stress of dislocation. The influence of SE on energy barrier and Peierls stress mainly results from the misfit energy and elastic strain energy become lower when the core of dislocation becomes wider. While the influence of elastic strain energy on energy barrier and Peierls stress mainly results from the cancellation between misfit energy and elastic strain energy for they possess comparable amplitudes but opposite phases. In addition, it is deduced that for the studied crystals, the shuffle dislocations control the deformation at medium and low temperatures, while glide partial dislocations are responsible for high temperature plasticity.

## Introduction

Among the semiconductor materials, the diamond-structured Si, sphalerite- structured GaAs, InP and CdTe have received widespread attention by reason of their important technical applications, for instance lasers, transistors, solar cells, optoelectronic devices, digital and spacecraft etc. [1–7]. Dislocations are ubiquitous in crystals. They have significant impacts on various properties which affect the performance and service life of materials [8, 9]. With the large-scale integration and increasing miniaturization of modern semiconductor devices, understanding the properties of dislocations is more and more important due to the defects, including dislocations, are the essential entities of device functions [10]. Core structure and Peierls stress are two essential quantities of dislocation for the plasticity of crystal has a close relationship to dislocation motion [11], and the movement of dislocation is associated with the core structure [12–14]. Therefore, for getting a better understanding of mechanical properties, so as to provide some useful information for improving the performance and prolonging the service life of related devices, investigating the structure and mechanical properties of dislocations in the related semiconductor materials has important implications.

Experimentally, the Peierls stresses of Si, GaAs, InP and CdTe have been estimated by Kamimura *et al*. by extrapolating the critical resolved shear stress to 0K directly [15]. Theoretically, except for the direct atomistic simulations, classical Peierls-Nabarro model (PN model) is the main approach for studying the fundamental properties of dislocations [16–18]. However, the quantitative agreement between the prediction and experimental result is not satisfactory. For improving the classical PN model, the force-balance equation of this model has been discretized and applied to evaluate the Peierls stress of several materials [19]. Although the treatment of discretization has effectively improved the estimated results, the estimated Peierls stresses for diamond- and sphalerite-structured crystals are still much higher than the experimental ones [15, 16, 19]. Recently, the lattice theory of dislocation(LTOD) has been developed by professor Wang, in which the SE missed in classical PN model is included and represented as the second derivative term of mismatch field in the force-balance equation [20–23]. In addition, the energy barrier and Peierls stress are calculated only from misfit energy *E*_*misfit*_ in classical PN model. While research suggests that the elastic strain energy *E*_*strain*_ cannot be ignored [24].

It is well known that for the dislocations in diamond- and sphalerite-structured crystals, the glide 60° dislocation dissociates into a 30° partial dislocation and a 90° partial dislocation separated by a ribbon of intrinsic stacking fault, and the glide screw dislocation dissociates into two 30° partial dislocations. While the shuffle dislocations do not dissociate due to there is no low energy stacking fault for a shuffle set. Experimentally, the slip of perfect $\langle 1\bar{1}0\rangle \left\{111\right\}$ dislocations in GaAs and InP have been observed in an intermediate temperature range under a confined high-pressure experiment [25]. For Si, the slip of perfect dislocation has also been observed at low temperatures [26]. While, there are no experimental reports on the sliding of perfect $\langle 1\bar{1}0\rangle \left\{111\right\}$ dislocations in CdTe. The screw dislocation generally possesses higher Peierls potential than the 60° dislocation. In addition, it has been established that high-temperature plasticity of diamond- and sphalerite-structured crystals is brought about by glide of dissociated dislocations on close packed planes, and the 30° partial generally possesses higher Peierls potential than the 90° partial, and consequently controls the deformation [15].

Therefore, in present paper, the structure and mechanical properties of glide 30° partial dislocations in Si, GaAs, InP and CdTe, and the shuffle screw dislocations in Si, GaAs and InP are studied based on the LTOD. Through comparing the calculated results with those given by classical PN model and experimental ones, the influences of SE and *E*_*strain*_ on structure and mechanical properties of the studied dislocations are systematically discussed.

## Theoretical model and determination of the factors in force-balance equation

The most famous theoretical model for investigating the basic properties of dislocation is classical PN model. In this model, for constructing a dislocation, the crystal is firstly cut along the misfit plane which becomes the surface layers. The surface plane and internal planes of the two semi-crystals resulted from division cannot be distinguished due to the continuum approximation. So the atomic interactions in surface plane, namely SE, is missed [27]. The SE has non negligible influences on dislocations, especially the narrow ones in covalent crystals. Consequently, the results of structure and mechanical property predicted by classical PN model are not satisfactory [16–18, 28]. For improving the classical PN model, Edagawa *et al*. have discretized the classical PN model based on the simplified version of the semi-discretized variational PN model(it will be called discretized PN model) [19]. Although the treatment of discretization has effectively improved the estimated result of Peierls stress, the SE is still missed.

In recent years, professor Wang has presented a general representation for dislocation, the LTOD [20–23]. In the LTOD, just as done in PN model, the crystal is divided into two semi-crystals along the glide plane of the dislocation. Due to nonlinear interaction, dislocation defect is produced when the two parts are glued non-trivially. According to the elastic interaction between atoms in each semi-crystal and the non-trivial interaction between two semi-crystals, the equilibrium equation of interaction forces can be determined. The unified equations possess the following form

$$-\frac{\alpha_{∥}}{2}\frac{d^{2}s_{∥}}{dx^{2}}-{\left.\frac{K_{∥}}{2\pi }\int_{-\infty }^{+\infty }\frac{dx\prime }{x\prime -x}\left(\frac{ds_{∥}}{dx}\right)\right|}_{x=x\prime }$$

$$+\frac{\tilde{\alpha }}{2}\frac{d^{2}s_{⊥}}{dx^{2}}+{\left.\frac{\tilde{K}}{2\pi }\int_{-\infty }^{+\infty }\frac{dx\prime }{x\prime -x}\left(\frac{ds_{⊥}}{dx}\right)\right|}_{x=x\prime }=f_{∥}$$ (1)

$$-\frac{\tilde{\alpha }}{2}\frac{d^{2}s_{∥}}{dx^{2}}-{\left.\frac{\tilde{K}}{2\pi }\int_{-\infty }^{+\infty }\frac{dx\prime }{x\prime -x}\left(\frac{ds_{∥}}{dx}\right)\right|}_{x=x\prime }$$

$$+\frac{\alpha_{⊥}}{2}\frac{d^{2}s_{⊥}}{dx^{2}}+{\left.\frac{K_{⊥}}{2\pi }\int_{-\infty }^{+\infty }\frac{dx\prime }{x\prime -x}\left(\frac{ds_{⊥}}{dx}\right)\right|}_{x=x\prime }=f_{⊥}$$ (2)

introducing *b* represents the Burgers vector, *s*_∥_ (*s*_⊥_) represents the displacement component parallel (perpendicular) to *b*. The definitions of restoring force components *f*_∥_ and *f*_⊥_ are similar to that of displacement component. *α*_∥_ (*α*_⊥_) and *K*_∥_ (*K*_⊥_) are the correction and energy factors parallel(perpendicular) to *b*. The correction and energy factors are given by [23]

$$\begin{array}{ll} \alpha_{∥}=\alpha_{E}\;{\text{sin}}^{2}\;\vartheta +\alpha_{S}\;{\text{cos}}^{2}\;\vartheta , & \alpha_{⊥}=\alpha_{E}\;{\text{cos}}^{2}\;\vartheta +\alpha_{S}\;{\text{sin}}^{2}\;\vartheta \\ K_{∥}=K_{E}\;{\text{sin}}^{2}\;\vartheta +K_{S}\;{\text{cos}}^{2}\;\vartheta , & K_{⊥}=K_{E}\;{\text{cos}}^{2}\;\vartheta +K_{S}\;{\text{sin}}^{2}\;\vartheta \\ \tilde{\alpha }=\left(\alpha_{E}-\alpha_{S}\right)\;\text{sin}\;\vartheta \;\text{cos}\;\vartheta , & \tilde{K}=\left(K_{E}-K_{S}\right)\;\text{sin}\;\vartheta \;\text{cos}\;\vartheta \end{array}$$

where *α*_*E*_ (*α*_*S*_) and *K*_*E*_(*K*_*S*_) are the correction factor and energy factor for edge (screw) dislocation, respectively. *ϑ* is the dislocation angle. Generally, *s*_⊥_ is regarded as a perturbation and can be neglected. And only the component *s*_∥_ is evaluated. The dislocation equation satisfied by *s*_∥_ can be approximately expressed as

$$-\frac{\alpha_{∥}}{2}\frac{d^{2}s_{∥}}{dx^{2}}-{\left.\frac{K_{∥}}{2\pi }\int_{-\infty }^{+\infty }\frac{dx\prime }{x\prime -x}\left(\frac{ds_{∥}}{dx}\right)\right|}_{x=x\prime }=f_{∥}$$ (3)

**Fig 1:**
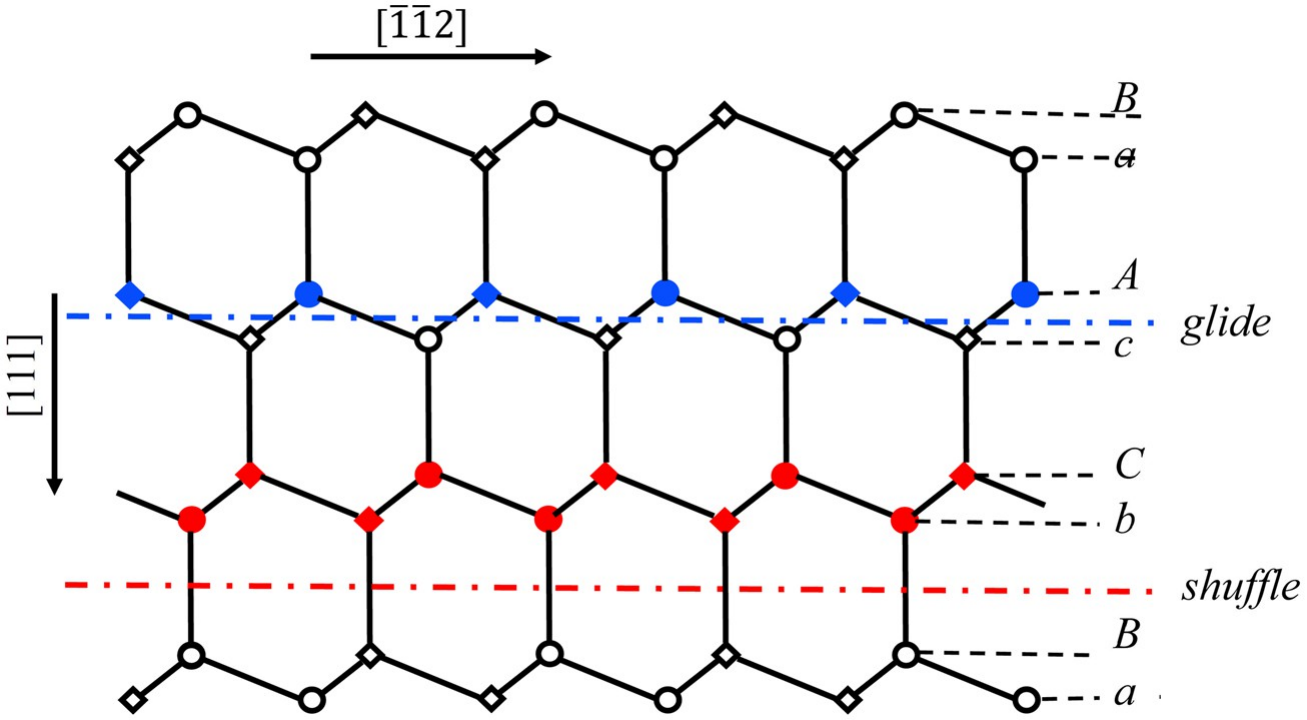
Two different sets of {111} planes for diamond- and sphalerite-structured crystals. The circle and quadrilateral symbols represent the atoms in and below the plane of paper, respectively.

The right side of dislocation Eq (3) represents the non-trivial interaction between two semi-crystals which is generally obtained from the negative gradient of the γ-surface. The left side represents the elastic interaction between atoms in a semi-crystal. The second-order differential term in left side is originated from the correction of SE which is resulted from the interaction between the atoms in surface plane [27]. As shown in Fig 1, the SE of glide dislocation mainly comes from the interaction between blues atoms, while that of shuffle dislocation mainly comes from the interaction between red atoms. The SE parameters *α*_∥_ for these two slip systems can be determined by a constructed statics model and represented by lattice and elastic constants [27].

For glide dislocations in diamond- and sphalerite-structured crystals, the correction factors *α*_*E*_ and *α*_*S*_ used for determining the SE parameter are respectively

$$\alpha_{E}=\frac{\left(C_{11}-C_{12}\right)a}{16},\;\;\;\alpha_{S}=0$$ (4)

and for shuffle dislocations

$$\alpha_{E}=\frac{\left(3C_{11}+5C_{12}\right)a}{24},\;\;\alpha_{S}=\frac{\left(C_{11}-C_{12}\right)a}{16}$$ (5)

The energy factors are

$$K_{E}=G\prime /\left(1-\nu \prime \right),\;\;K_{S}=G\prime$$ (6)

Introducing $\left\{C_{ij}^{\prime }\right\}$ represents the elastic-coefficient matrix from the symmetry of crystal relative to the new reference axes (a rotation of 45° about the ***i*** axis of the cube axes), *G*′ and *ν*′ can be obtained by the following equations [29]

$$G\prime =\sqrt{C_{44}^{\prime }C_{55}^{\prime }}$$ (7)

$$\nu \prime =\frac{\tilde{C}-G\prime }{\tilde{C}}$$ (8)

with

$$\tilde{C}=\frac{1}{3}\left(2+\frac{C_{22}^{\prime }}{{\bar{C}}_{11}^{\prime }}\right)\left({\bar{C}}_{11}^{\prime }+C_{12}^{\prime }\right)\sqrt{\frac{C_{55}^{\prime }\left({\bar{C}}_{11}^{\prime }-C_{12}^{\prime }\right)}{C_{22}^{\prime }\left({\bar{C}}_{11}^{\prime }+C_{12}^{\prime }+2C_{55}^{\prime }\right)}}$$

where ${\bar{C}}_{11}^{\prime }=\sqrt{C_{11}^{\prime }C_{22}^{\prime }}$, the relations between elements $C_{ij}^{\prime }$ and elastic constants *C*_*ij*_ are

$$C_{11}^{\prime }=C_{11},\;\;C_{12}^{\prime }=C_{12},\;C_{55}^{\prime }=C_{44}$$

$$C_{22}^{\prime }=C_{11}+\frac{H}{2},\;C_{44}^{\prime }=C_{44}-\frac{H}{2},\;H=2C_{44}+C_{12}-C_{11}$$

where *H* is the anisotropy factor.

The restoring force *f*_∥_ which is used for closing the equation of dislocation is generally obtained by *f*_∥_(*s*) = −∇*γ*(*s*) [30]. The *γ*-surface and restoring force can be expressed as follows

$$\gamma \left(s\right)=\Sigma_{i}\gamma_{i}\;{\text{cos}}^{2i}\left(\frac{\pi s}{b}\right)$$ (9)

$$f_{∥}\left(s\right)=\frac{2\pi }{b}\;\text{sin}\left(\frac{\pi s}{b}\right)\Sigma_{i}i\gamma_{i}\;{\text{cos}}^{2i-1}\left(\frac{\pi s}{b}\right)$$ (10)

where *γ*_*i*_ is the fitting parameter of *γ*-surface.

The nonlinear integro-differential force-balance Eq (3) can be hardly solved exactly. In present paper, the truncating method proposed by Wang is used to solve the equation [23, 31]. The trial solution of force-balance Eq (3) can be expressed as

$$s_{∥}\left(x\right)=s_{∥}^{0}+s_{∥}^{c}$$ (11)

with

$$s_{∥}^{0}=\frac{b}{\pi }\text{arctan}p,\;p=\kappa x$$ (12)

$$s_{∥}^{c}=\frac{bp}{\pi }\left[\lambda_{1}w_{1}\left(y\right)+\lambda_{2}w_{2}\left(y\right)+\cdots \right]$$ (13)

where $s_{∥}^{0}$ and $s_{∥}^{c}$ mainly describe the overall profile and the core structure, respectively. *λ*_*m*_, the value of *m* takes the natural number greater than zero, is the core parameter of dislocation needs to be determined. *y* = 1/(1 + *p*^2^), and *w*_*m*_ is a set of orthogonal polynomials whose expression is as follows

$$w_{m}\left(y\right)=\Sigma_{l=1}^{m}g_{l}^{m}y^{l}$$ (14)

with

$$g_{l}^{m}=\frac{{\left(-1\right)}^{m+l}\left(m+l\right)!}{\left(l-1\right)!\left(l+1\right)!\left(m-l\right)!}$$ (15)

the field of definition for the orthogonal polynomials is (0,1). Substituting the expressions of $s_{∥}^{0}$ and $s_{∥}^{c}$ into Eq (11), the full expression of the trial solution can be obtained

$$s_{∥}\left(x\right)=\frac{b}{\pi }\left[\text{arctan}p+\lambda_{1}pw_{1}\left(y\right)+\lambda_{2}pw_{2}\left(y\right)+\cdots \right]$$ (16)

The complexity of calculation increases rapidly with the increasing of the number of core parameters. For different dislocations, the trial solution Eq (16) can be truncated at a suitable position. In diamond- and sphalerite-structured crystals, even for the dissociated dislocation, its two partials can be treated as isolated ones due to the low intrinsic stacking fault energy (ISFE). Consequently, retaining one term in $s_{∥}^{c}$ is enough to describe the dislocations studied in present paper. In this case, the parameter *κ* is

$$\kappa =\frac{2\lambda }{d}{\left(\frac{{\text{sin}}^{2}\;\vartheta }{1-\nu \prime }+{\text{cos}}^{2}\;\vartheta \right)}^{-1}$$

where *d* represents the spacing between two atomic layers of misfit plane. Core parameter *λ* = *λ*_1_ can be determined by substituting the trial solution into force-balance equation and solving the equation by truncating method. After substituting, one needs to determine the results of the differential and integral terms on left side of the equation, and expand the trigonometric functions in restoring force into power series and truncate it at a suitable position. Both sides can be written as the polynomials of *y* with a pre-factor *p*. Then integrate the polynomials in the field of definition (0,1) and solve the equation to obtain the core parameter *λ*. If neglecting the SE (the SE parameter *α*_∥_ = 0), Eq (3) degenerates into classical PN model.

## The core parameter and core structure

Core structure is an important quantity of dislocation due to the motion of dislocation is closely related to it. Core width represents the range of severe lattice distortion area caused by dislocation, so it is an important parameter of core structure. The core width is characterized by the coverage distance of *x* when *s*(*x*) changes from −*b*/4 to *b*/4. It can be easily calculated when the core parameter *λ* is determined. In order to calculate the parameter *λ*, the SE parameter *α*_∥_, energy factor *K*_∥_ in dislocation Eq (3) and the *γ*-surface which is necessary for obtaining the restoring force should be determined firstly. The constants used for determining *α*_∥_, *K*_∥_ and the key parameters needed in calculation are listed in Table 1.

**Table 1:** Key quantities for studying dislocations in Si, GaAs, InP and CdTe. The lattice constant *a* and mechanical parameters are respectively in units of Å and GPa.

Crystals	*a*	*C*_11_	*C*_12_	*C*_44_	*G*′	*v*′	*G*	*ν*	*G*′/*G*	*ν*′/*ν*
**Si**	5.43^a^	165.7^b^	63.9	79.6	63.7	0.257	68.1^b^	0.218^b^	0.94	1.18
**GaAs**	5.65^c^	119.0^c^	53.8	59.5	44.0	0.291	32.6^c^	0.31^c^	1.35	0.94
**InP**	5.87^d^	102.2^e^	57.6	46.0	32.0	0.351	22.5^h^	0.3^i^	1.42	1.17
**CdTe**	6.48^f^	53.5^g^	36.5	19.9	13.0	0.415	8^j^	0.338^k^	1.63	1.23

^a^Ref. [17],

^b^Ref. [29],

^c^Ref [32],

^d^Ref [33],

^e^Ref [34],

^f^Ref [35],

^g^Ref [36],

^h^Ref [37],

^i^Ref [38],

^j^Ref [39],

^k^Ref [40]

**Fig 2:**
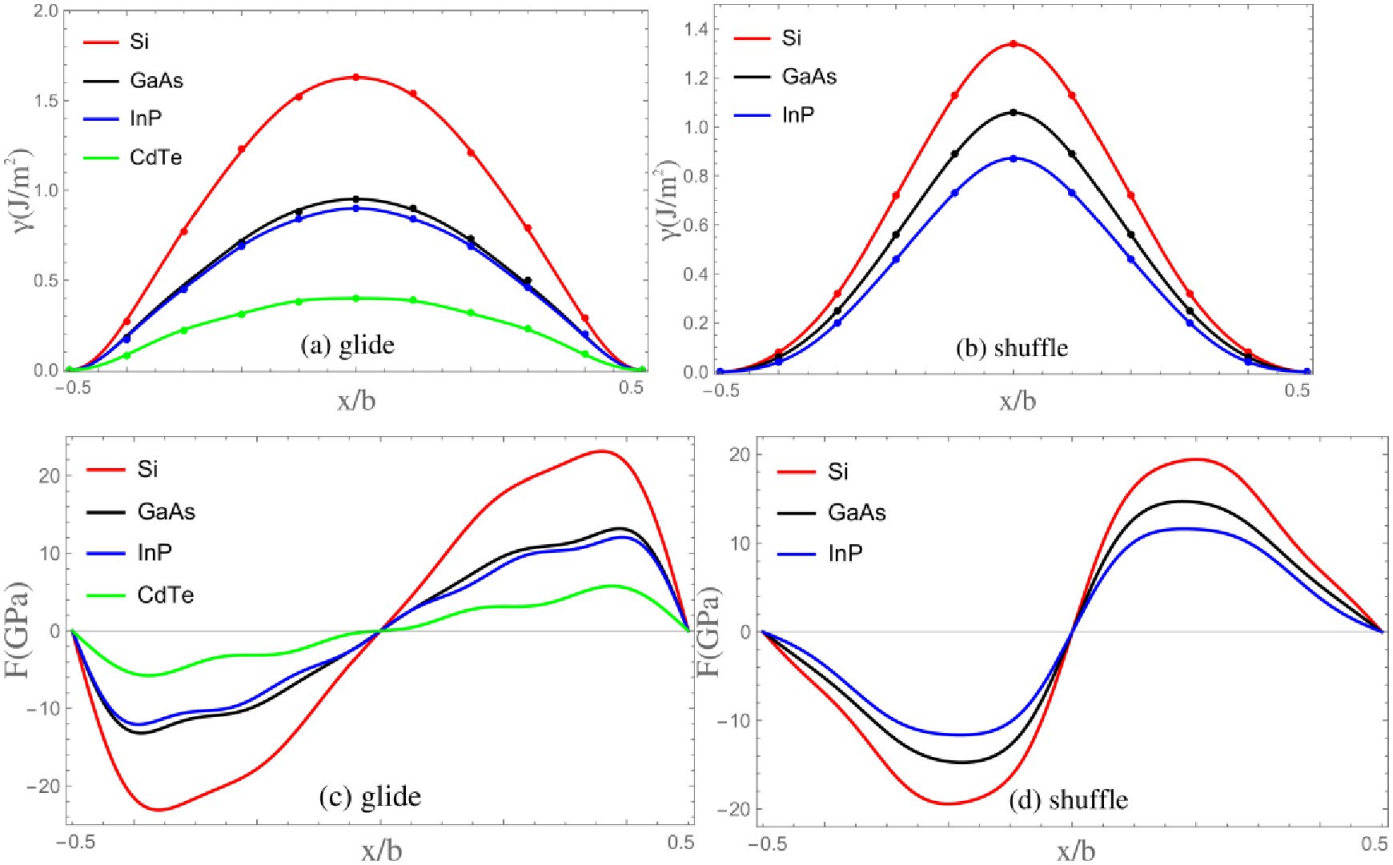
The *γ*-surface and restoring force. Discrete points represent the numerical results.

The γ-surfaces in Ref. [16] have been used to investigate the mobility of dislocations in Si, zinc-blende crystals GaAs, InP and CdTe by Kamimura *et al*. [16] and Edagawa *et al*. [19], respectively. To compare the calculated results with theirs, the same γ-surfaces are used in present calculations. The *γ*-surfaces along 1/6〈112〉 of glide set, 1/2〈110〉 of shuffle set are plotted in Fig 2. The corresponding restoring force are plotted in the meanwhile. Fitting parameters of γ-surface are given in Table 2.

**Table 2:** The fitting parameters *γ*_*i*_ in unit of J/m^2^.

Dislocation	Crystal	*γ*_1_	*γ*_2_	*γ*_3_	*γ*_4_	*γ*_5_
**30° partial**	Si	3.324	-4.58	5.255	-2.882	0.512
	GaAs	2.412	-5.152	8.271	-6.628	2.048
	InP	2.392	-5.64	10.058	-8.982	3.071
	CdTe	0.99	-1.012	-0.43	1.877	-1.025
**shuffle screw**	Si	0.864	-0.575	3.481	-4.478	2.047
	GaAs	0.612	0.053	1.327	-1.957	1.023
	InP	0.342	0.851	-0.373	-0.461	0.513

**Fig 3:**
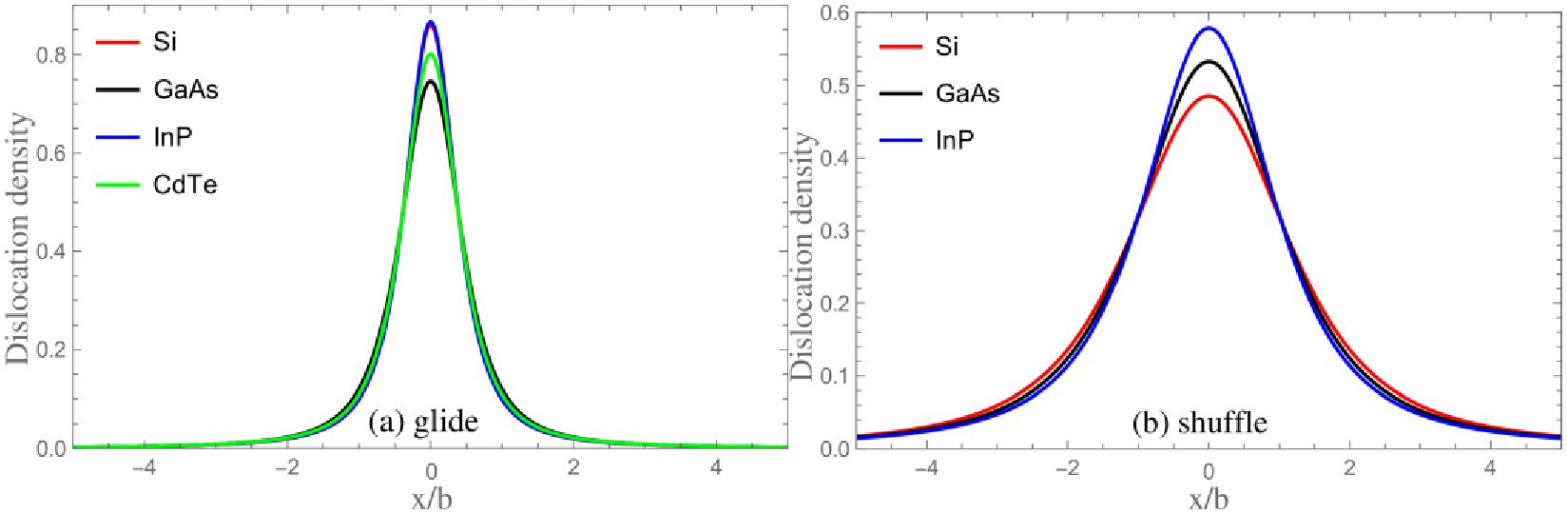
The maps of dislocation densities obtained from LTOD.

**Fig 4:**
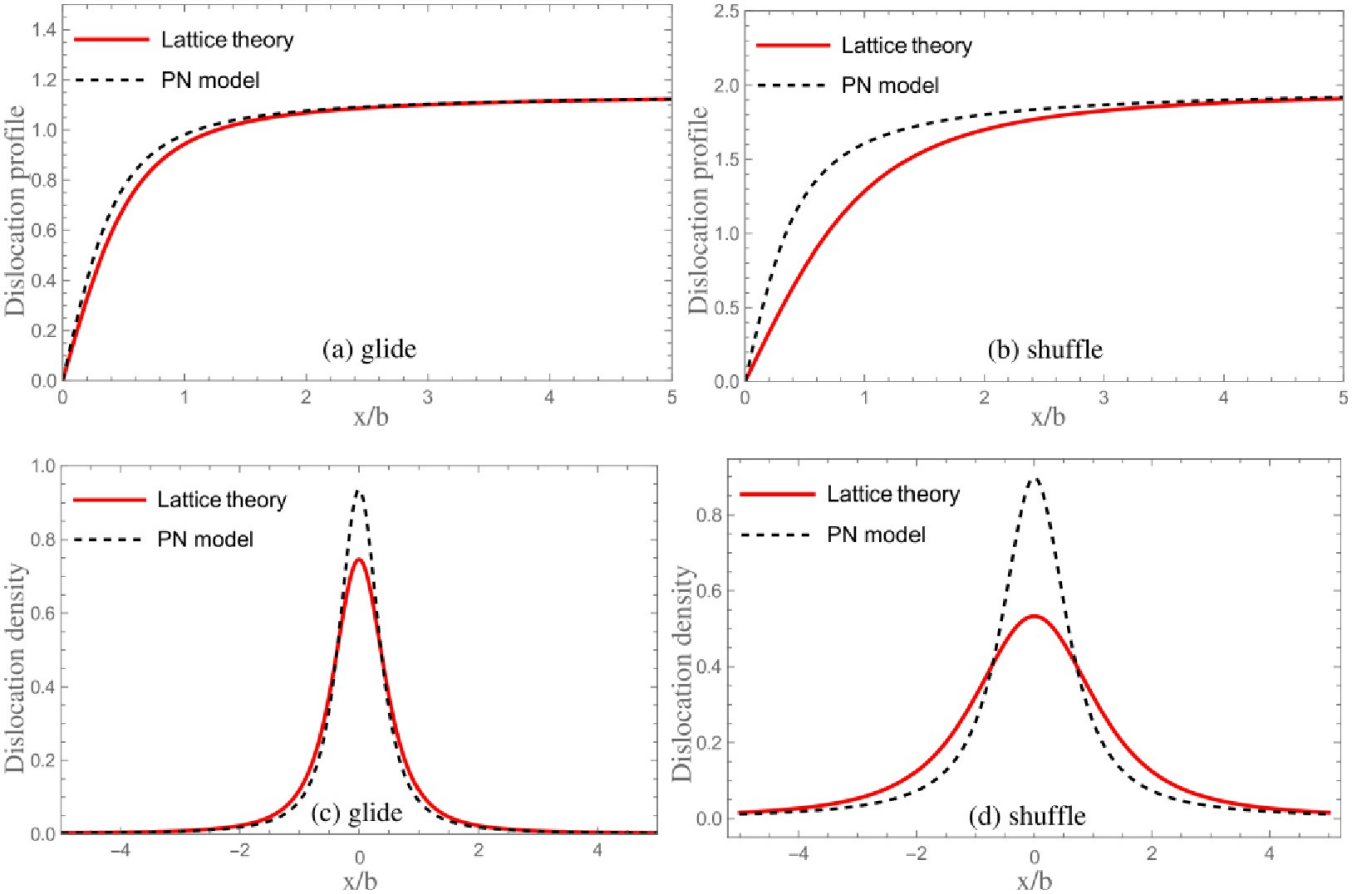
The comparison of dislocation profiles/densities for GaAs obtained by classical PN model and LTOD.

According to the truncating method, the core parameter *λ* and core width *ζ* are calculated. Meanwhile, the results of *λ*_*PN*_ and *ζ*_*PN*_ obtained from neglecting SE are also given in Table 3. The dislocation densities *ρ*(*x*) = *ds*(*x*)/*dx* are shown in Fig 3. For purpose of showing influence of SE on core structure more intuitively, in Fig 4, the comparison of dislocation profiles (densities) for GaAs obtained by classical PN model and LTOD is shown. One can see that the dislocation is widened after considering the SE. This is mainly because the stronger elastic interaction on the left side of Eq (3) will lead to dislocation be widened, while the stronger interaction between two semi-crystals(the higher unstable stacking fault energy(USFE)) on the right side of Eq (3) will lead to dislocation be narrowed. After considering the SE, the elastic interaction becomes stronger, and therefore, the core width becomes wider. Researches show that the movement of dislocation is associated with the core structure: the wider dislocation corresponds to lower Peierls stress [21, 41]. Therefore, it is inferred that the result of Peierls stress after considering SE should be lower than that calculated by the PN model. In addition, as shown in Fig 1, the SE of shuffle and glide dislocations come from the interactions between the red and blue atoms in {111} plane, respectively. The adjacent red atoms interact directly through covalent bonds, which is stronger than the interaction between blue atoms. Therefore, the correction of SE to shuffle dislocation is more obvious.

**Table 3:** The results of *λ* and *ζ* calculated from LTOD. *λ*_*PN*_ and *ζ*_*PN*_ are the results of neglecting the SE. *ζ*_*PN*_ and *ζ* are in unit of Å.

Dislocation	Crystal	*λ*_*PN*_	*λ*	*ζ*_*PN*_	*ζ*_*PN*_/*b*	*ζ*	*ζ/b*	*ζ*/*ζ*_*PN*_
**30° partial**	Si	0.553	0.694	1.15	0.52	1.50	0.68	1.30
	GaAs	0.654	0.737	1.45	0.63	1.79	0.78	1.23
	InP	0.558	0.674	1.30	0.54	1.62	0.67	1.25
	CdTe	0.597	0.690	1.59	0.60	1.92	0.73	1.21
**shuffle screw**	Si	0.332	0.730	2.92	0.76	4.58	1.19	1.57
	GaAs	0.371	0.772	2.72	0.68	4.36	1.09	1.60
	InP	0.491	0.785	2.75	0.66	4.20	1.01	1.53

## The energy barrier and Peierls stress

The research shows that in addition to the *E*_*misfit*_ used for calculation of Peierls stress in the classical PN model, there exists another energy, the *E*_*strain*_, will affect the calculated results of energy barrier and Peierls stress and cannot be ignored in calculation [24]. In the present study, the Peierls stress is calculated from total energy in which both the contributions of the two energies just mentioned are considered. The Hamiltonian consists of three parts

$$H=H_{1}+H_{2}+H_{12}$$ (17)

where *H*_12_ is the Hamiltonian of the interaction between two semi-crystals, *H*_1_ (*H*_2_) describes the approximated harmonic interactions between atoms in each semi-crystal. The interactions are expressed as

$$H_{12}=\Sigma_{j}\gamma \left(s_{j}\right)$$ (18)

$$H_{1}=H_{2}=\frac{1}{2}\Sigma_{j}F_{j}\cdot s_{j}$$ (19)

**Fig 5:**
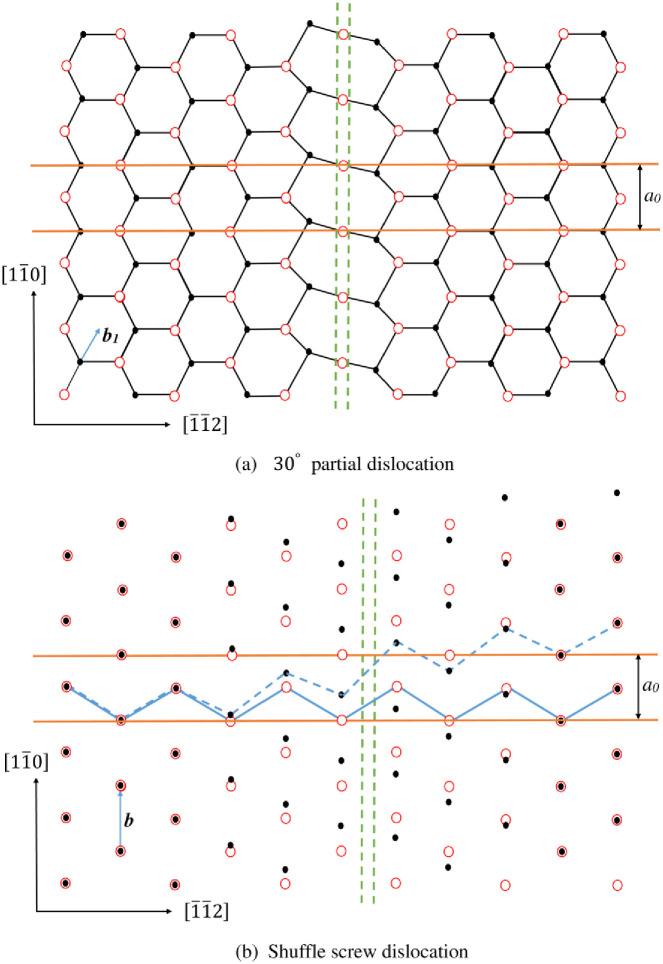
The projection of the dislocation in glide plane. The black and red symbols respectively stand for atoms in and below the plane of paper. The deformation is represented by relative displacement of black circles for simplicity. The distance along *b* between dotted line and solid line in (b) represents relative displacement.

***F*** is the harmonic interaction force between atoms in each semi-crystal. The sums run over the atoms on which a non-zero force is applied. Consequently, the *E*_*misfit*_ and *E*_*strain*_ of dislocation per unit length are

$$E_{misfit}\left(x_{0}\right)=\frac{H_{12}}{L}=\frac{\sigma }{a_{0}}\Sigma_{k=-\infty }^{+\infty }\gamma \left(s_{k}\right)$$ (20)

$$E_{strain}\left(x_{0}\right)=\frac{1}{L}\left(H_{1}+H_{2}\right)=\frac{\sigma }{2a_{0}}\Sigma_{k=-\infty }^{+\infty }F\left(s_{k}\right)s_{k}$$ (21)

where *a*_0_ is the period along the center line of dislocation. *x*_0_ and *L* respectively represent the position and length of dislocation, *s*_*k*_ = *s*(*x*_*k*_ − *x*_0_) represents the displacement of atom located at *x*_*k*_. The sums cover the area with width *a*_0_ in Fig 5. The energies per unit length are

$$E_{misfit}\left(x_{0}\right)=\frac{\sigma }{a_{0}}\Sigma_{k=-\infty }^{+\infty }\gamma \left\{s\left[\frac{3\left(2n+\delta \right)}{4}b-x_{0}\right]\right\}$$ (22)

$$E_{strain}\left(x_{0}\right)=\frac{\sigma }{2a_{0}}\Sigma_{k=-\infty }^{+\infty }F\left\{s\left[\frac{3\left(2n+\delta \right)}{4}b-x_{0}\right]\right\}s\left[\frac{3\left(2n+\delta \right)}{4}b-x_{0}\right]$$ (23)

$$E_{total}\left(x_{0}\right)=E_{misfit}\left(x_{0}\right)+E_{strain}\left(x_{0}\right)$$ (24)

A parameter *δ* is introduced due to if there are atoms on the symmetry axis of a free stable dislocation is undetermined. *δ* = 0 corresponds to there exists atoms on the symmetry axis, and conversely, *δ* = 1 corresponds to there exists no atoms.

**Fig 6:**
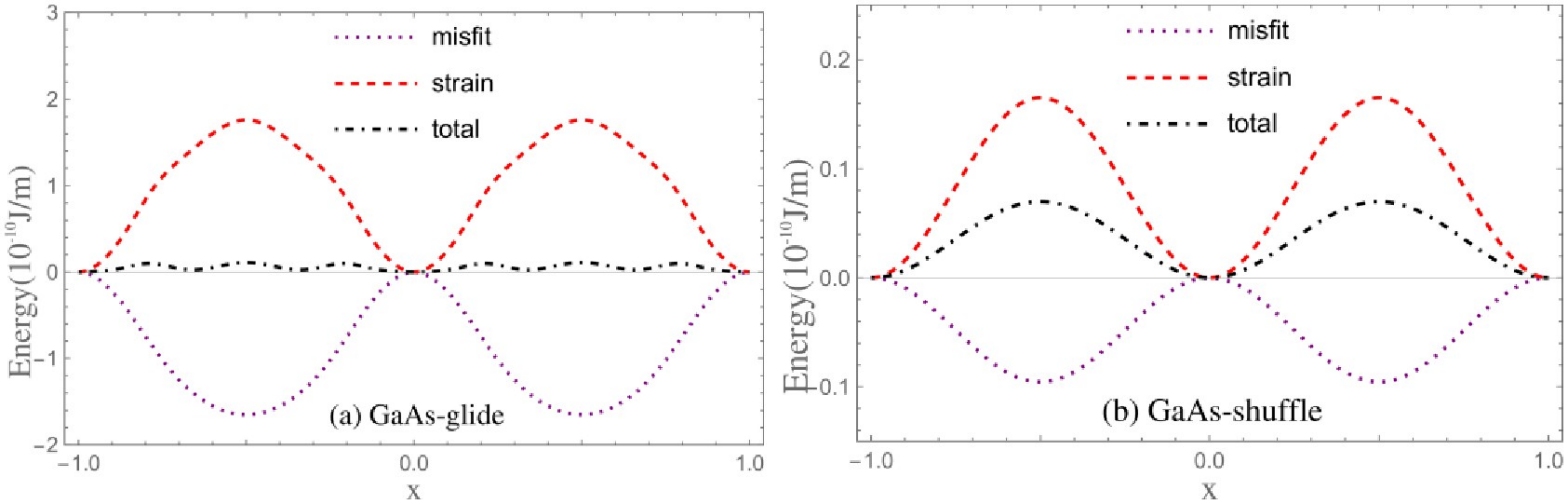
The energy curves for GaAs.

For the dislocation in diamond- and sphalerite-structured crystals, except for an additional constant that does not affect the calculated result, the summation in the formula of energy converges rapidly due to the dislocation is very narrow. The relationship between energy and dislocation position is calculated. It is found that for same type dislocation, the two cases of *δ* values correspond to the highest and lowest energy states, respectively. Based on minimum energy principle, for the free stable 30° partial and shuffle screw dislocations, the center line of the former coincides with a row of atoms on the mismatch plane, while that of the latter lies between two rows of atoms(just as shown in Fig 5). For the studied crystals, except for the energy barriers are different, the shapes of energy curves are similar for same type dislocations, hence only the energy curves of GaAs are plotted in Fig 6. It can be seen that *E*_*misfit*_ and *E*_*strain*_ possess opposite phases and change periodically when the dislocation moves. Their amplitudes are comparable. For 30° partial dislocation, the energy valleys appear between the nearest neighbor ground states on account of the competition between *E*_*misfit*_ and *E*_*strain*_. The new energy valleys imply that there exists metastable states aside from the ground state.

Peierls stress can be calculated by

$$\tau_{P}=\frac{1}{b}\text{max}\left|\frac{dE_{total}}{dx}\right|$$ (25)

If *α*_∥_ and *E*_*strain*_ equal to zero, the calculated result is that of classical PN model. The calculated results see Table 4.

**Table 4:** The calculated results of Peierls stress in unit of GPa. Where subscripts *p* and *PN* correspond to the results of considering and neglecting the SE, respectively. The superscript *m* corresponds to consider *E*_*misfit*_ only, and *m* + *s* corresponds to consider *E*_*misfit*_ *and E*_*strain*_ simultaneously. *τ*^*exp*^ is the experimental result taken from Ref. [15] except that of Si is taken form Ref [42]. *τ*^*C*−*PN*^ and *τ*^*D*−*PN*^ are the results of Ref. [16] and Ref. [19], respectively.

Dislocation	Crystals	*τ* ^*C*−*PN*^	*τ* ^*D*−*PN*^	$\tau_{PN}^{m}$	$\tau_{PN}^{m+s}$	$\tau_{p}^{m}$	$\tau_{p}^{m+s}$	*τ* ^*exp*^
**30° partial**	Si	45.1	11.3	22.1	7.7	16.4	5.5	4.64±1.39
	GaAs	23.0	5.9	9.6	4.3	7.2	3.9	3.21±0.96
	InP	25.2	6.5	10.0	4.7	7.8	3.8	2.21±0.66
	CdTe	10.5	2.8	3.5	2.4	2.8	1.0	0.57±0.40
**shuffle screw**	Si	-	-	3.5	0.56	0.91	0.35	1.5
	GaAs	4.14	2.7	2.6	0.30	0.38	0.28	3.00±0.90
	InP	3.10	2.1	2.1	0.26	0.24	0.21	1.60±0.48

**Fig 7:**
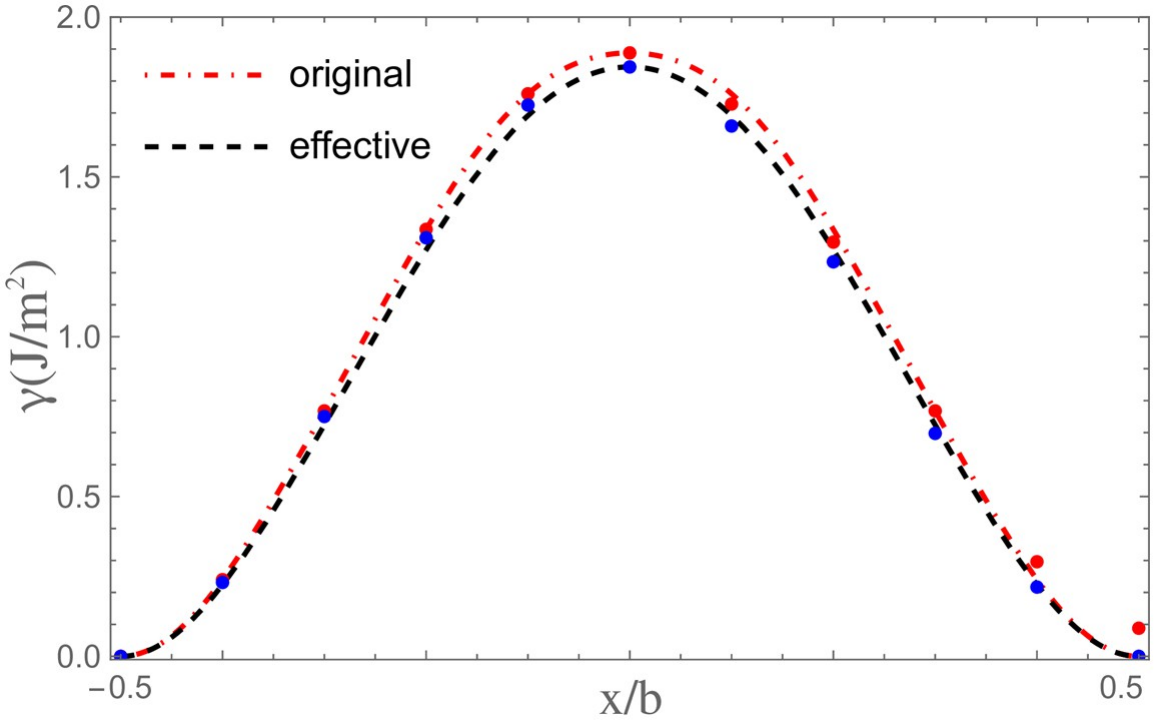
The original *γ*-surface for glide set of Si given by Joos *et al*. and its corresponding effective *γ*-surface.

Theoretically, when *α*_∥_ and *E*_*strain*_ equal to zero, the result of $\tau_{PN}^{m}$ given by present paper should be basically identical with *τ*^*C*−*PN*^ estimated by classical PN model. However, the calculated $\tau_{PN}^{m}$ is about one half to one third of *τ*^*C*−*PN*^ given by Ref. [16] for 30° partial dislocations, and about two-thirds of *τ*^*C*−*PN*^ for shuffle screw dislocations. The reasonableness of $\tau_{PN}^{m}$ and *τ*^*C*−*PN*^ should be ascertained firstly. Consequently, $\tau_{PN}^{m}$ for Si is calculated with the *γ*-surface given by Joos *et al*. and compared with the result given by them using the classical PN model [17]. Effective *γ*-surface obtained from the original one given by Joos *et al*. is also used for calculation to investigate the difference between Peierls stresses calculated with original and effective *γ*-surfaces. The used *γ*-surfaces are shown in Fig 7, the results of estimated Peierls stresses see Table 5. One can see that the result of $\tau_{PN}^{m}$ is about 36.3GPa, it is basically identical with the 35.9GPa given by Joos *et al*. When effective *γ*-surface given in Ref. [16] is used, the result of $\tau_{PN}^{m}$ is 22.1GPa, about one half of the 45.1GPa calculated by Kamimura *et al*. Generally, the level of USFE directly affects the magnitude of Peierls stress. The USFE given by Kamimura *et al*. is 1642 mJ/m^2^ [16], it is smaller than the 1888 mJ/m^2^ given by Joos *et al*. The Peierls stress given by Ref. [16] should be smaller than that given by Joos *et al*. Besides, the tiny difference between two *γ*-surfaces resulted from the extremely low ISFE relative to USFE makes minute and negligible difference between corresponding Peierls stresses.

**Table 5:** The fitting parameters of the original and effective *γ*-surfaces for 30° partial dislocation in Si, and the calculated Peierls stress. The Peierls stress and fitting parameters are in units of GPa and J/m^2^, respectively. The result calculated by Joos *et al*. using classical PN model is also shown in parenthesis.

Used *γ*-surface	*γ*_1_	*γ*_2_	*γ*_3_	*γ*_4_	*γ*_5_	$\tau_{PN}^{m}$	$\tau_{PN}^{m+s}$	$\tau_{p}^{m}$	$\tau_{p}^{m+s}$
**Original**	2.660	-1.619	0.841	0.826	-0.819	36.3(35.9)	11.9	26.5	7.77
**Effective**	2.490	-1.685	1.923	-1.089	0.205	34.8	11.4	25.2	7.42

**Fig 8:**
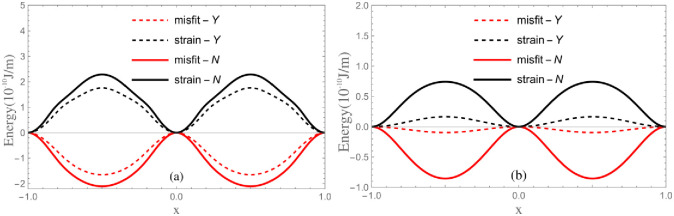
The influence of SE on *E*_*misfit*_ and *E*_*strain*_. The thick and dashed lines correspond to the results without and with considering the SE, respectively.

The Peierls stress $\tau_{p}^{m+s}$ predicted by LTOD is much smaller than that estimated by classical PN model. Through comparing $\tau_{p}^{m}$ and $\tau_{PN}^{m}$, we can see that $\tau_{p}^{m}$ is decreased after considering SE. This is mainly because narrower core means higher degree of mismatch which corresponds to the higher *E*_*misfit*_ and *E*_*strain*_. After considering the SE, the dislocation becomes wider, consequently, *E*_*misfit*_ and *E*_*strain*_ become lower(see Fig 8). Therefore, when the Peierls stress is calculated from *E*_*misfit*_ only, the result of $\tau_{p}^{m}$ given by LTOD is lower than $\tau_{PN}^{m}$ given by PN model. This is consistent with the previous inference based on the dislocation width situation. One can see that the result of $\tau_{p}^{m}$ is closer to the experimental result, therefore, the core width obtained by considering the SE is more reasonable. When the Peierls stress is calculated from *E*_*total*_, it can be seen from Figs 6 and 8 that the *E*_*misfit*_ and *E*_*strain*_ possess comparable amplitudes but opposite phases. Therefore, compared to the Peierls stress calculated from *E*_*misfit*_ only, the result calculated from *E*_*total*_ is greatly decreased whether considering the SE or not. Through comparing the results of $\tau_{p}^{m+s}$ and $\tau_{PN}^{m+s}$, it is found that when both *E*_*misfit*_ and *E*_*strain*_ are taken into account, the Peierls stress calculated by considering the SE is lower. It means that the decrease of the barrier of *E*_*misfit*_ and *E*_*strain*_ which results from SE generally makes the Peierls stress be lowered. It should be pointed out that the reduction of Peierls stress after considering SE is not simply determined by the *E*_*misfit*_ or *E*_*strain*_, but by the relative value of these two energies. As mentioned in the previous section, for shuffle dislocation, the SE mainly comes from the interactions between the first nearest neighbor atoms, while comes from the interactions between second nearest neighbor atoms for glide dislocation(see Fig 1). Therefore, the correction of SE to shuffle screw dislocation is more obvious. Besides, compared with $\tau_{PN}^{m}$, the results of *τ*^*D*−*PN*^ given by Edagawa et al. [19] using discretized PN model are decreased effectively and become closer to experimental results. However, comparing with $\tau_{PN}^{m+s}$, *τ*^*D*−*PN*^ is still larger. This indicates that the correction of *E*_*strain*_ is larger than discretization. Through comparing the results of ${\;\tau }_{PN}^{m}$, *τ*^*D*−*PN*^ and ${\;\tau }_{p}^{m}$, it is found that the correction of discretization to Peierls stress is more obvious than that of SE. Actually, both discretization and SE are resulted from the discreteness of lattice. Compared to discretization, SE can be considered as the higher-order correction.

Experimentally, it is found that in the low temperature region below 300~400°C to room temperature, the yield strength of Si is about 1.5GPa [43]. Rabier group reported the existence of perfect dislocations in Si deformed at low temperature at a stress of ~1GPa [44, 45]. The yield strength values of GaAs determined from compression experiments at room temperature is less than 1GPa [25, 46] It is worth noting that the results of *τ*^*exp*^ in Table 4 are the estimated results for 0K from the experimental data. In addition, because of the influence of temperature is not considered in calculation, the calculated results correspond to the case of 0K. One can see that the calculated results of Peierls stress for 30° partial dislocations in Si and GaAs are larger than the yield stress at low temperature, while those for shuffle screw dislocations are less than but close to the yield stress. Furthermore, the results of 30° partial dislocations estimated by LTOD is consistent with the results which are extrapolated to 0K from the experimental data at high temperatures. Therefore, it is deduced that shuffle dislocations control the deformation at medium and low temperatures, while glide partial dislocations are responsible for high temperature plasticity.

## Summary and discussion

In calculation, four cases are taken into account, such as neglecting both SE and the contribution of *E*_*strain*_ simultaneously, neglecting SE but including the contribution of *E*_*strain*_, neglecting the contribution of *E*_*strain*_ but including SE, including both SE and the contribution of *E*_*strain*_. The first case corresponds to classical PN model, and the last case is LTOD. It can be found that, despite the same *γ*-surface is used, the result of $\tau_{PN}^{m}$ calculated by present paper and that of *τ*^*C*−*PN*^ given by Kamimura *et al*. [16] are inconsistent. By discussing the 30° partial in Si and according to the general relationship between USFE and Peierls stress, it is thought that the $\tau_{PN}^{m}$ calculated by present paper is more reasonable. Besides, for researching the influences of using original and effective γ-surfaces in calculation on the results of estimation, the effective γ-surface obtained from the original one given in Ref. [17] is also used for calculation of Peierls stress. It is found that for material whose ISFE is much lower than USFE, the difference of the results calculated with these two *γ*-surfaces is not obvious and negligible. Consequently, one can use the original *γ*-surface directly in calculation. For shuffle dislocation, the SE is mainly contributed by the interactions between nearest neighbor atoms, while for glide partial dislocation, it is mainly contributed by the interactions between second nearest neighbor atoms. Hence the correction of SE to shuffle screw dislocation is more obvious. The *E*_*strain*_ possesses opposite phase and comparable amplitude with *E*_*misfit*_, therefore, the Peierls stress calculated by *E*_*misfit*_ is much larger than that calculated by *E*_*total*_. Through comparing the calculated results with those given by Kamimura *et al*. [16] and Edagawa *et al*. [19], it can be found that the result of Peierls stress estimated by present paper is more consistent with the experimental date, therefore, LTOD is more reliable in theoretical estimation of Peierls stress. According to the similarity of diamond- and sphalerite-structured crystals, it is deduced that shuffle dislocations control the deformation at medium and low temperatures, while glide partial dislocations are responsible for high temperature plasticity.

## Conclusion

The basic properties of dislocations in Si, GaAs, InP and CdTe are investigated based on the LTOD. The research indicates that the elastic interaction on left side of the dislocation equation becomes stronger when SE is considered, therefore, the core width of dislocation becomes wider. Compared to glide partial dislocation, the correction of SE to shuffle dislocation is more obvious. Both the SE and the *E*_*strain*_ affect the energy barrier and Peierls stress of dislocation. The influence of SE on the energy barrier and Peierls stress mainly results from *E*_*misfit*_ and *E*_*strain*_ become lower when the core width of dislocation becomes wider. While the influence of *E*_*strain*_ on energy barrier and Peierls stress mainly results from the cancellation between *E*_*strain*_ and *E*_*misfit*_ for their comparable amplitudes but opposite phases. By comparing the calculated results with the yield strength measured experimentally and the Peierls stress at 0K extrapolated from the experimental data, it is deduced that for the studied crystals, the shuffle dislocations control the deformation at medium and low temperatures, while glide partial dislocations are responsible for high temperature plasticity.
